# Supplementary material for: Possible Association between Bladder Wall Morphological Changes on Computed Tomography and Bladder-Centered Interstitial Cystitis/Bladder Pain Syndrome
Source: Biomedicines. 2021 Sep 24;9(10):1306. doi: 10.3390/biomedicines9101306 (PMC8533058; doi:10.3390/biomedicines9101306)
Supplement: Supplementary file 1 [file biomedicines-09-01306-s001.zip › biomedicines-1359857-supplementary.pdf]

The criteria for including IC/BPS patient in current study:

1. Patients meets the ESSIC criteria for IC/BPS (chronic (> 6 months) pelvic pain, pressure, or discomfort perceived to be related to the urinary bladder accompanied by at least one other urinary symptom such as persistent urge to void or frequency)
2. The IC/BPS patients who were refractory to oral medication treatments.
3. The IC/BPS patients who were admitted to our hospital for cystoscopic hydrodistention
4. The IC/BPS patients agreed to join this study and provide informed consent.

The criteria for excluding patients in current study:

1. The patients with concurrent urological diseases such as bacterial cystitis or urolithiasis
2. The patients who had been previously diagnosed as neurogenic voiding dysfunction, eosinophilc cystitis, urothelial cancer or ketamine-related cystitis
3. The patients with benign or malignant tumor in cystoscopy.
4. The patients who had undergone urological procedures in recent 6 months, such as cystoscopic hydrodistention or intravesical instillation of any therapeutic agent.

Histopathological grading definitions:

inflammatory cell infiltration grades 0, 1, 2, and 3 were based on <10, 10–50, 50–100, >100 inflammatory cells/HPF in the submucosal area, respectively. Eosinophil and plasma cell infiltration was based on >10 cells/HPF in the submucosal area
